# A Concurrent Pseudoaneurysm and an Arteriovenous Fistula Following Percutaneous Radial Artery Access

**DOI:** 10.7759/cureus.31207

**Published:** 2022-11-07

**Authors:** Ryan J Bolt, Syed Sadiq M Jafri, Thomas S Siegel, Steven Werns

**Affiliations:** 1 General Surgery, Beaumont Health Dearborn Hospital, Dearborn, USA; 2 Cardiology, Beaumont Health Dearborn Hospital, Dearborn, USA

**Keywords:** color flow doppler ultrasound, radial arterial catheter, cardiac cathetarization, arteriovenous fistula repair, radial artery pseudoaneurysm

## Abstract

The incidence of radial artery cannulation resulting in the concurrent development of a pseudoaneurysm and an arteriovenous fistula is not well defined. Here, we present the case of a 42-year-old man who developed an iatrogenic pseudoaneurysm (PSA) and a concurrent arteriovenous fistula (AVF) following multiple right radial artery cannulations. Access was obtained for a preoperative diagnostic cardiac catheterization and again for hemodynamic monitoring intraoperatively during a surgical aortic valve replacement. A palpable thrill over the right radial artery developed and persisted for nine months, leading to anxiety and mental fixation on the thrill. There were no other symptoms. Given a failed resolution with conservative care for the same duration, the patient elected to proceed with surgical resection. Following resection, the patient reported resolution of his symptoms and decreased anxiety. A follow-up targeted arterial ultrasound demonstrated no residual PSA or AVF.

## Introduction

Radial artery cannulation is a common practice for close hemodynamic monitoring in critical care and intraoperative settings. Additionally, transradial access for cardiac catheterizations is now the preferred approach worldwide. Advantages of radial access over femoral access include reduced bleeding, even in the setting of antiplatelet and anticoagulation use; lower complication rates; a decreased length of stay; and increased patient satisfaction [1]. Patient satisfaction with the radial artery approach was shown in the Radial vs. Femoral (RIVAL) trial. The RIVAL trial found that over 90% of patients expressed a preference for repeating the transradial approach if needed, compared to 49% of patients who desired to repeat the transfemoral approach [2]. Complications, while rare, included radial artery spasm, non-coronary artery dissection, perforation, occlusion, arteriovenous fistula, pseudoaneurysm, compartment syndrome, and hand ischemia [3].

## Case presentation

**Figure 1:**
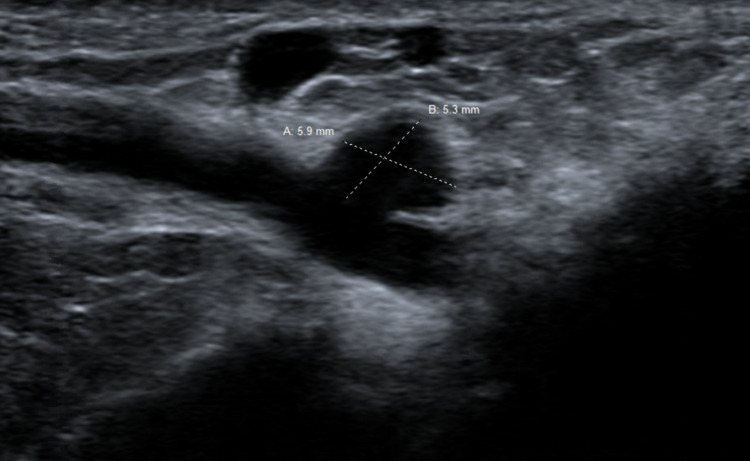
A pseudoaneurysm measuring 5.9 mm x 5.3 mm

**Figure 2:**
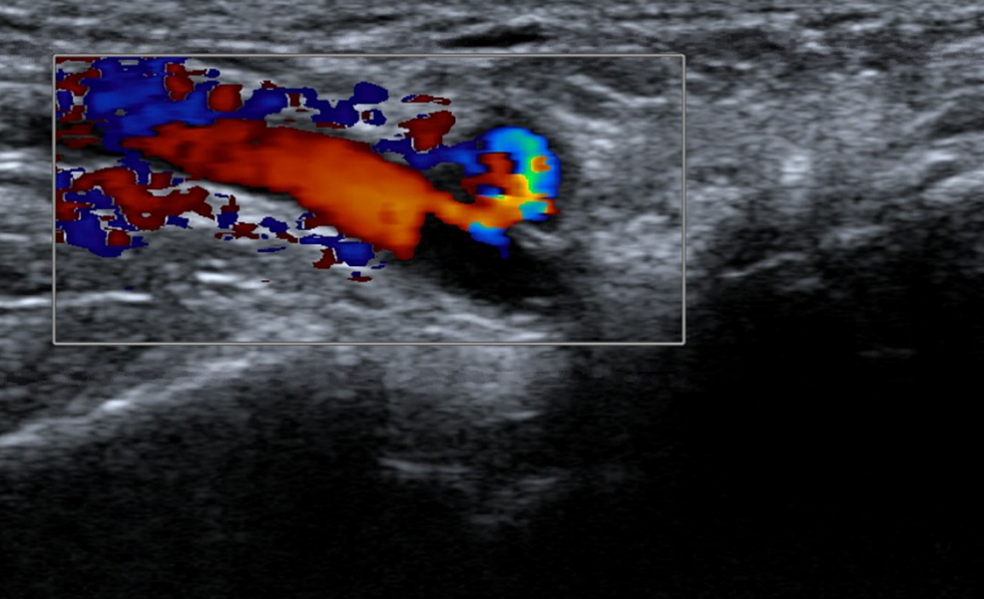
Radial artery pseudoaneurysm with a turbulent flow

A 42-year-old male with a history significant for a bicuspid aortic valve with severe aortic stenosis underwent a diagnostic cardiac catheterization with access via the right radial artery for preoperative evaluation before undergoing a surgical aortic valve replacement (SAVR). His catheterization was uncomplicated and revealed normal coronary arteries, and he subsequently had the SAVR performed three months later. On presentation for SAVR, an arterial line was placed in the right radial artery for close hemodynamic monitoring in the perioperative period. His postoperative course was uncomplicated. On follow-up in the cardiology clinic, he was noted to have a palpable thrill on exam of his right radial pulse as well as an audible bruit with auscultation. Motor and sensory function remained normal. He was referred for vascular surgical evaluation, where physical exam findings were confirmed. Ultrasound was performed, showing a right radial pseudoaneurysm (PSA) with a turbulent flow (Figures 1- 2).

**Figure 3:**
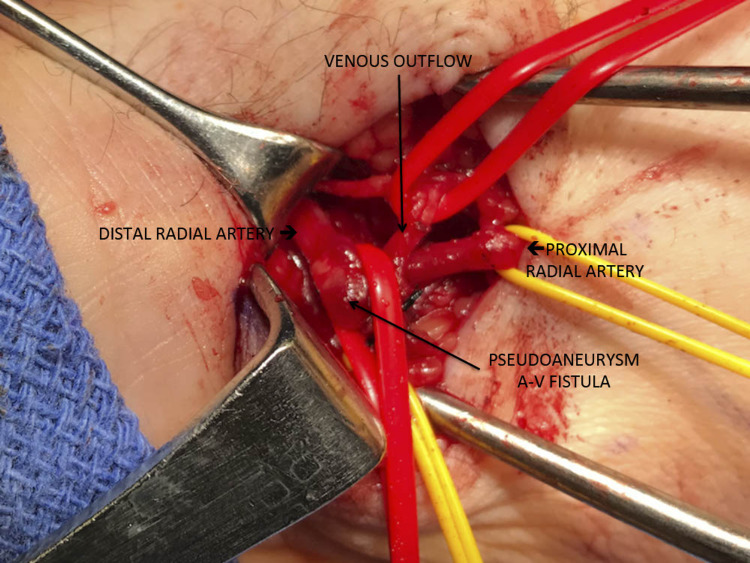
A radial artery pseudoaneurysm with an arteriovenous fistula (intraoperative)

Conservative management for nine months was undertaken; however, the PSA did not resolve. The patient was scheduled for surgical resection. Intraoperatively, he was found to have an arteriovenous fistula (AVF) with concurrent PSA, which was successfully ligated and resected (Figure 3).

Dissection began overlying the site of palpable thrill about the right radial artery and was carried down through subcutaneous tissues. Proximal and distal control of the radial artery was obtained. The venous contribution to the AVF was isolated and ligated with 3-0 silk. Following this, the pseudoaneurysm was resected, and the radial artery was repaired with a continuous 6-0 Prolene. After the case, a strong distal radial pulse was present, confirmed by palpation and Doppler. During the postoperative follow-up, continued resolution was confirmed by repeat arterial ultrasound, demonstrating no residual PSA or AVF.

## Discussion

Arteriovenous fistula (AVF) is defined as an anatomically abnormal communication between an artery and vein [4]. AVFs can be classified as congenital or acquired, in the form of iatrogenic or traumatic. The most common site of iatrogenic AVF is the common femoral artery (incidence of 37%), most commonly following diagnostic procedures [1]. Upper extremity AVFs resulting from percutaneous access of the radial and ulnar arteries for cardiac interventions are a rare complication, with an incidence of 0.02% to 0.04% of cases [4].

A pseudoaneurysm (PSA) is defined as an outpouching of an artery that does not include all arterial wall layers. Adventitia of the artery or a rim of fibrous tissue may compose the PSA wall [5]. The most common cause of a PSA is iatrogenic. However, there are other causes, which include trauma (penetrating or blunt), infection, vasculitis, or spontaneous causes. Percutaneous access is the most common cause of iatrogenic PSA [5]. Risk factors contributing to the increased likelihood of PSA development include multiple puncture attempts, catheter infection, anticoagulation, vascular site infection, and large sheath size [3, 5].

Radial artery PSA coupled with AVF formation is a rare complication, with an incidence that is not well defined. Isolated PSA or AVF formations are more common yet remain exceedingly rare (0.1% for PSA and 0.02-0.04% for AVF) [1, 4]. Our case included at least two separate cannulations in a three-month time frame, which may have contributed to our patient's complication. Additional undocumented attempts at percutaneous access prior to cannulation that were not successful cannot be excluded. It is also unclear if cannulation was attempted with ultrasound guidance.

Frequently, these complications can be treated conservatively without requiring surgical intervention. PSAs often resolve with prolonged manual compression, correction of coagulopathy, or with thrombin injection [3, 6]. AVFs rarely require surgical intervention and can be treated with transradial compression bands and expectant waiting [4].

The surgical indications for the resection of PSAs and AVFs are similar. Indications for PSAs include symptoms (i.e., pain), failure to resolve with conservative measures, or a diameter >3cm [3]. Indications for AVFs include limb ischemia and high-output heart failure [7].

## Conclusions

In patients with concurrent iatrogenic PSA and AVF, operative management can be an effective treatment option following failed conservative management. Furthermore, the incidence and recommended management of such pathology are not well-defined and may warrant further investigation.

## References

[1] Bhatt DL (2015). Vascular access and closure. Cardiovascular Intervention: A Companion to Braunwald’s Heart Disease.

[2] Jolly SS, Niemelä K, Xavier D (2011). Design and rationale of the radial versus femoral access for coronary intervention (RIVAL) trial: a randomized comparison of radial versus femoral access for coronary angiography or intervention in patients with acute coronary syndromes. Am Heart J.

[3] Riangwiwat T, Blankenship JC (2021). Vascular complications of transradial access for cardiac catheterization. US Cardiol Rev.

[4] Sidawy A, Perler B (2018). Acquired arteriovenous fistulas. Rutherford’s Vascular Surgery and Endovascular Therapy.

[5] Cameron JL, Cameron AM (2019). Pseudoaneurysms and arteriovenous fistulas. Current Surgical Therapy.

[6] Kongunattan V, Ganesh N (2018). Radial artery pseudoaneurysm following cardiac catheterization: a nonsurgical conservative management approach. Heart Views.

[7] Tatli E, Buturak A, Cakar A (2015). Unusual vascular complications associated with transradial coronary procedures among 10,324 patients: case based experience and treatment options. J Interv Cardiol.

